# The objectives and instructional design of undergraduate endodontic program: multicenter cross-sectional study in Saudi Arabia

**DOI:** 10.1186/s12909-022-03548-8

**Published:** 2022-06-23

**Authors:** Fahda N. Algahtani, Reem M. Barakat, Rahaf A. Almohareb, Lujain Alqarni, Alanoud Alqabbani, Ebtisam Almadi

**Affiliations:** 1 Department of Clinical Dental Sciences, College of Dentistry, Princess Nourah bint Abdulrahman University, P.O. Box 84428, Riyadh, 11671 Saudi Arabia; 2 Dental Intern, College of Dentistry, Princess Nourah bint Abdulrahman University, P.O. Box 84428, Riyadh, 11671 Saudi Arabia; 3 Department of Restorative Dental Science, College of Dentistry, King Saud University, Riyadh, Saudi Arabia

**Keywords:** Dental education, Endodontics, Dental student, Dental school, Endodontic curriculum

## Abstract

**Background:**

Identify the objectives and the instructional design of undergraduate endodontics in dental schools in Saudi Arabia.

**Methods:**

The online questionnaire was developed from an original survey conducted in the United Kingdom. The questionnaire was modified for purpose of the study and the region of interest. Then it was directed and emailed to the undergraduate endodontic program directors in twenty-six dental schools in Saudi Arabia. The results were analyzed using descriptive statistics and the Chi-square and Fisher’s exact tests.

**Results:**

The response rate was 96.15%. The number of credit hours for preclinical endodontic courses was up to four credit hours (84%). Students were clinically trained to do vital pulp therapies (92%), root canal treatment (100%), and root canal retreatment (68%). The majority of dental schools define the minimum clinical requirements (92%). Practical and clinical competency exams were used to evaluate students' performance (92% and 84% respectively). The students were trained to treat cases of minimal (52%) to moderate complexity (48%). Endodontic treatment consent and difficulty assessment form were used by 32% and 60% of dental schools respectively. There was no significant difference in the instructional design between public and private dental schools (*P* > 0.05).

**Conclusion:**

The endodontic undergraduate objectives were to graduate competent clinicians who acquired basic science of endodontics and who know their limitations as it is necessary for a safe general dental practice. The use of endodontic treatment consent and case difficulty assessment should be wisely considered in clinical training.

**Supplementary Information:**

The online version contains supplementary material available at 10.1186/s12909-022-03548-8.

## Background

Periodontium health is an essential component of overall body health [1–3]. Root canal treatment (RCT) has proven to be effective in restoring the health of the supporting periodontium in non-vital teeth [4–6]. Moreover, endodontic treatment can help prevent the development of apical lesions in vital cases [5–7].

General dentists are often the first-line practitioners who provide endodontic interventions for patients in need [8, 9]. Unfortunately, the quality of RCT in cross-sectional studies is often described as inadequate or substandard [10–14]. Possible causes were the complexity of root canal procedures or the graduation of inexperienced practitioners [15, 16]. Fortunately, the introduction of modern technologies, instruments, and materials such as nickel-titanium rotary files and bioceramic sealers made RCT procedures simpler and more predictable for general practitioners [15, 17–21].

Ideally, the undergraduate student should master case selection, become competent in using modern techniques for RCT, and have adequate clinical experience before graduation. Thankfully, these educational objectives are permissible when international treatment standards and guidelines are being followed in a well-designed dental curriculum. The Commission of Dental Accreditation (CODA), the Association of Dental Education in Europe (ADEE), and the European Society of Endodontology (ESE) recommended competency-based education in a comprehensive clinical care environment [15, 22, 23]. Furthermore, an endodontic difficulty assessment form or tool has been developed to help guide students and general dentists in case selection [24–26].

The Ministry of Education outlines the national qualification framework for the dental specialty in Saudi Arabia. This framework was made to meet the demand for nationally and internationally qualified Saudi dental graduates [27]. Saudi dental graduates are expected to provide high-quality services inside the country and complete their studies or work abroad. Competency-based education is integral to the academic curriculum because it measures and verifies the attainment of intended program learning outcomes [28]. Finally, dental graduates have to commit to a lifelong journey of continuing education and meet the requirements for Saudi licensure registration [29]. However, studies found that Saudi students were not confident in doing steps of RCT or in managing endodontic emergencies [30–32]. A similar finding was also reported internationally which signifies the importance of well-designed undergraduate endodontic curricula [33–35].

Knowledge about the quality of undergraduate endodontic education for dental practitioners in Saudi Arabia is obscure due to the absence of national observational studies. Therefore, this study aimed to investigate the endodontic course directors' main objectives from undergraduate endodontic education in dental schools of Saudi Arabia. Furthermore. The study will explore the instructional design of endodontic education that course directors followed to meet their objectives.

## Material and method

The study was exempt from ethical approval by the institutional review board at Princess Nourah Bint Abdulrahman University (IRB log number: 20–016). The questionnaire was developed from a previous survey made in the United Kingdom [36] and modified to suit the region of interest and the study objectives. The modified questionnaire was written in the English language and piloted on a convenient sample of endodontic faculty. Comments about wording sentences, abbreviations, and punctuations were reviewed and discussed. The suggested changes were considered in the final version of the questionnaire.

The online questionnaire (SurveyMonkey, Momentive, CA, USA) started with a clarification of study objectives, assurance of confidentiality, and consent for participation. The first question was the type of school sector, followed by a question about the program director's objectives in the undergraduate endodontic program. There were eleven questions related to the instructional design of endodontic education (Table 1). All the questions required an answer before complete submission. The newly developed questionnaire was internally piloted and tested for readability and modified accordingly.

**Table 1:** The qestionnaire

General	● Please select your college/university's main sector or alliance (government sector, private sector)
Objective	● In a few statements. Mention the main aim of undergraduate endodontic education in your school?
Instructional design	1- Please select the credit hours^a^ for preclinical courses. (4 credit hours or less, 5 or 6 credit hours, 7 or 8 h, more than 8 credit hours)
	2- Which of these topics are taught in the didactic teaching of the endodontic program? Check all applicable (root canal anatomy and pulp histology, pulp pathology and endodontic microbiology, endodontic radiology, endodontic materials, vital pulp therapies, root canal treatment on immature teeth with non-vital pulp tissue, root canal treatment, root canal re-treatment, endodontic surgery, endodontic regeneration, restoration of root-filled teeth, bleaching of endodontically treated teeth, dental trauma, endodontic emergency, other (please specify))
	3- What are the types of root canals used in the laboratory setting? Check all applicable (canals in natural teeth, canals in plastic teeth available commercially, canals in acrylic blocks with simple curves, canals in acrylic blocks with S-shaped curve, 3D printed teeth with canals)
	4- Select the teeth that undergraduate students are allowed to treat in clinics. Check all applicable (incisors and canines, premolars, first molar, second molar)
	5- Which type of treatments are allowed to be performed by students during pre-clinical/clinical endodontic training? Choose all applicable (vital pulp therapies including pulp capping and pulpotomy, root canal treatment, root canal re-treatment, endodontic surgery, treatment of teeth with open apices, pulp regeneration.)
	6- Is there any definition for a minimum number of teeth clinically required to be completed before graduation? (yes, no)
	7- if yes, please indicate the number of teeth or canals in each applicable category: Incisors or single canal system (comment space), premolars or two canals system ( comment space), molars or three to four canals system (comment space), other (comment space)
	8- Is there any practical competency exam? Select the applicable. (preclinical: anterior, premolar, molar, none) (clinical: anterior, premolar, molar, none)
	9- Are students using a treatment consent that is specific for endodontics? (Yes, a special endodontic consent form is present, the tx consent is embedded in the general dental tx consent, verbal consent, and mutual agreement)
	10- Do you follow a difficulty assessment form before assigning clinical cases to students? (yes, the American Association of Endodontics (AAE) difficulty assessment form, yes, there is a difficulty assessment form. (not AAE form), no special difficulty assessment form)
	11- Select the level of case complexity that students are trained to do in clinics using the AAE difficulty assessment form (cases of minimal difficulty, cases of moderate difficulty, cases of high difficulty)

^a^ One credit hour is equal to 15 contact hours of lecture or direct instruction time. Also, one credit hour is equal to 30 contact hours of laboratory/preclinical simulation time

A list of all the public and private dental schools in Saudi Arabia was obtained from the Ministry of Education, and the emails of the Deans were obtained from the Saudi Dental Education Society. The Deans were initially contacted with the objectives of the study and to obtain their consent for participation. Then they were requested to email the questionnaire to the undergraduate endodontic program director, who will answer the questions on behalf of the dental school. The responses were analyzed using JMP statistical software (SAS Institute, NC, USA) and the data were summarized using descriptive statistics. The Chi-square and Fisher’s exact tests were used to statically determine the relationship between the collected categorical data and the type of dental school. The level of significance was *P* ≤ 0.05.

## Results

Twenty-five out of the twenty-six dental schools answered the questionnaire resulting in a 96.15% response rate. The number of participating public dental schools was 18 (72%) and seven private dental schools (28%). The program directors' objectives of undergraduate endodontic education were collectively summarized into three main objectives. The first objective is to acquaint students with the basic science of endodontics such as histology, microbiology, pathology, anatomy, and radiology. The second objective was to prepare a dental graduate who is competent in endodontic diagnosis, treatment planning, and management of simple root canal treatments, traumatic dental injuries, and emergencies. They also emphasized that every graduate is aware of the importance of recall and knows how to evaluate the success of their intervention. The third objective was to follow endodontic international standards and guidelines in preparing graduates, and equip them with the ability to recognize the limitation of their skills and experience in managing difficult cases and the necessity of referral in these situations.

The answers to several instructional design questions and their statistical association to the type of dental school are presented in Table 2. The students spend four credit hours or less in preclinical endodontic training (84%), where they are trained using natural teeth (96%). In the clinical setting, students are allowed to mainly treat anterior teeth (100%), premolars (100%), and first molars (92%). Only 36% of the programs train students to treat second molars. Moreover, students are trained to do vital pulp therapy and root canal treatment. However, none of the schools allow students to do apical surgery and only two schools train students to do pulp regeneration. Few schools require students to use a special endodontic treatment consent (32%) and the same applies to using the endodontic difficulty assessment form (40%) (Table 2). Students are allowed to treat simple and moderately difficult cases only.

**Table 2:** The instructional design of undergraduate endodontics in Saudi dental schools and the relationship between the collected categorical data and the type of dental school

The topic	The answer	Dental schools n (%)	Dental school type n		Public versus private schools (*P*-value)
			Public	Private	
Credit hours for preclinical courses	4 or less	21 (84)	14	7	*P* = 0.66
	5 or 6	1 (4)	1	0	
	7 or 8	0 (0)	0	0	
	More than 8	3 (12)	3	0	
Types of root canals used in the pre-clinic training	Canals in natural teeth	24 (96)	17	7	*P* = 0.38
	Canals in plastic teeth available commercially	12 (48)	11	1	
	3D printed teeth with canals	4 (16)	3	1	
	Canals in acrylic blocks with simple curves	2 (8)	2	0	
	Canals in acrylic blocks with an S-shaped curve	0 (0)			
Type of teeth allowed to be treated in the clinic	Incisors and canine	25 (100)	18	7	*P* = 0.54
	Premolars	25 (100)	18	7	
	1^st^ molar	23 (92)	18	5	
	2^nd^ molar	9 (36)	6	3	
Treatments allowed in the clinic	Vital pulp therapies	23 (92)	17	6	*P* = 0.82
	Root canal treatment	25 (100)	18	7	
	Root canal re-treatment	17 (68)	12	5	
	Endodontic surgery	0 (0)	0	0	
	Treatment of immature teeth	5 (20)	2	3	
	Pulp regeneration	2 (8)	1	1	
Minimum dental requirements	Yes	23 (92)	17	6	*P* = 0.49
	No	2 (8)	1	1	
Practical competency exam (preclinical)	Yes	23 (92)	15	7	*P* = 0.80
	No	2 (8)	2	0	
Clinical competency test	Yes	21 (84)	14	7	*P* = 0.54
	No	2 (8)	2	0	
	No response	2 (8)	2	0	
The use of treatment consent	Special endodontic treatment consent	8 (32)	7	1	*P* = 1.00
	Embedded in the general dental tx consent	12 (48)	7	5	
	Verbal consent and mutual agreement	5 (20)	4	1	
The use of difficulty assessment form	AAE difficulty assessment form	12 (48)	11	1	*P* = 0.37
	A difficulty assessment form. (not AAE form)	3 (12)	1	2	
	No special difficulty assessment form	10 (40)	6	4	
The level of case complexity	Cases of minimal difficulty	13 (52)	11	2	*P* = 0.21
	Cases of moderate difficulty	12 (48)	7	5	
	Cases of high difficulty	0 (0)	0	0	

Statistical significance was set on *P* ≤ 0.05

All of the undergraduate endodontic programs cover the following topics in their teaching: Root canal anatomy and pulp histology (100%), pulp pathology and endodontic microbiology (100%), endodontic radiology (100%), endodontic materials (100%), root canal treatment (100%). Moreover, the majority of dental schools are teaching vital pulp therapy (84%), root canal treatment of immature teeth with necrotic pulp (88%), root canal re-treatment (88%), endodontic surgery (84%), endodontic regeneration (80%), restoration of root-filled teeth (92%), bleaching of endodontically treated teeth (72%), dental trauma (84%), endodontic emergency (92%). There are four dental schools (16%) that mentioned other topics such as endodontic diagnosis and differential diagnosis, case selection and treatment planning, visual aids for diagnosis, endo-perio relationship, resorption, endodontic pain management, rotary files, magnification, and endodontic advancement. The Chi-square and Fisher’s exact tests reveled no significant assosiations between the type of the instructional design and the type of dental schools (*P* > 0.05).

The majority of the dental schools (92%) required students to pass a preclinical practical competency before commencing clinical training. The students have to pass a preclinical root canal treatment competency on anterior teeth (36%), premolars (32%), and molars (24%) before the beginning of clinical training. Four dental schools mentioned that the preclinical competency exam was both on anterior and premolar teeth. One school conducted three preclinical competency exams using plastic teeth, one on an anterior tooth, premolar, and molar. For clinical competency exams, most schools performed them on either anterior teeth (56%), premolars (44%), or both (16%). Clinical competency exams on molars were less emphasized compared to other teeth types (28%). One school stated that their competency exam involved asking the student to complete root canal treatment of a single canal system in one visit.

**Fig. 1:**
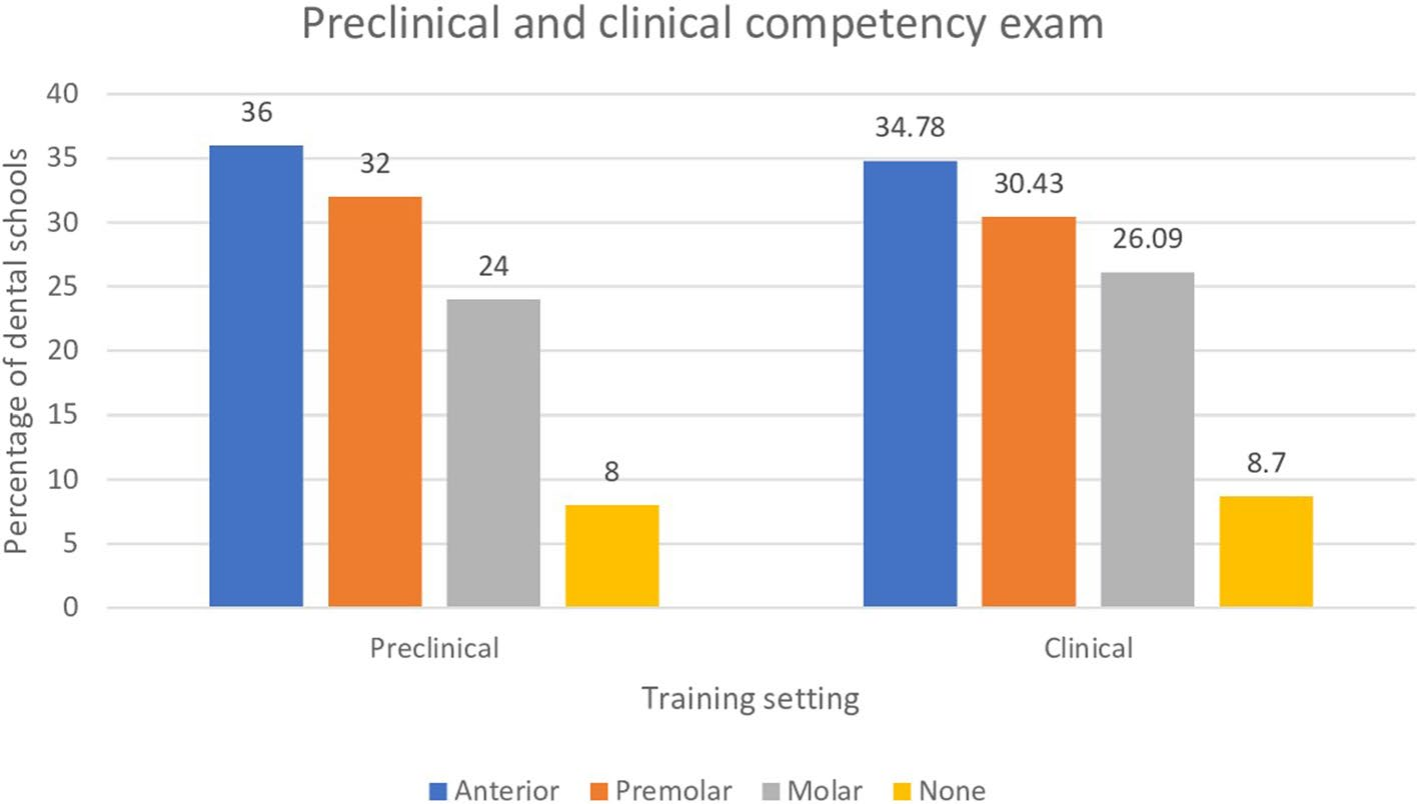
Percentage of dental schools that use anterior, premolar and molar teeth in competency exams in preclinical and clinical training

**Fig. 2:**
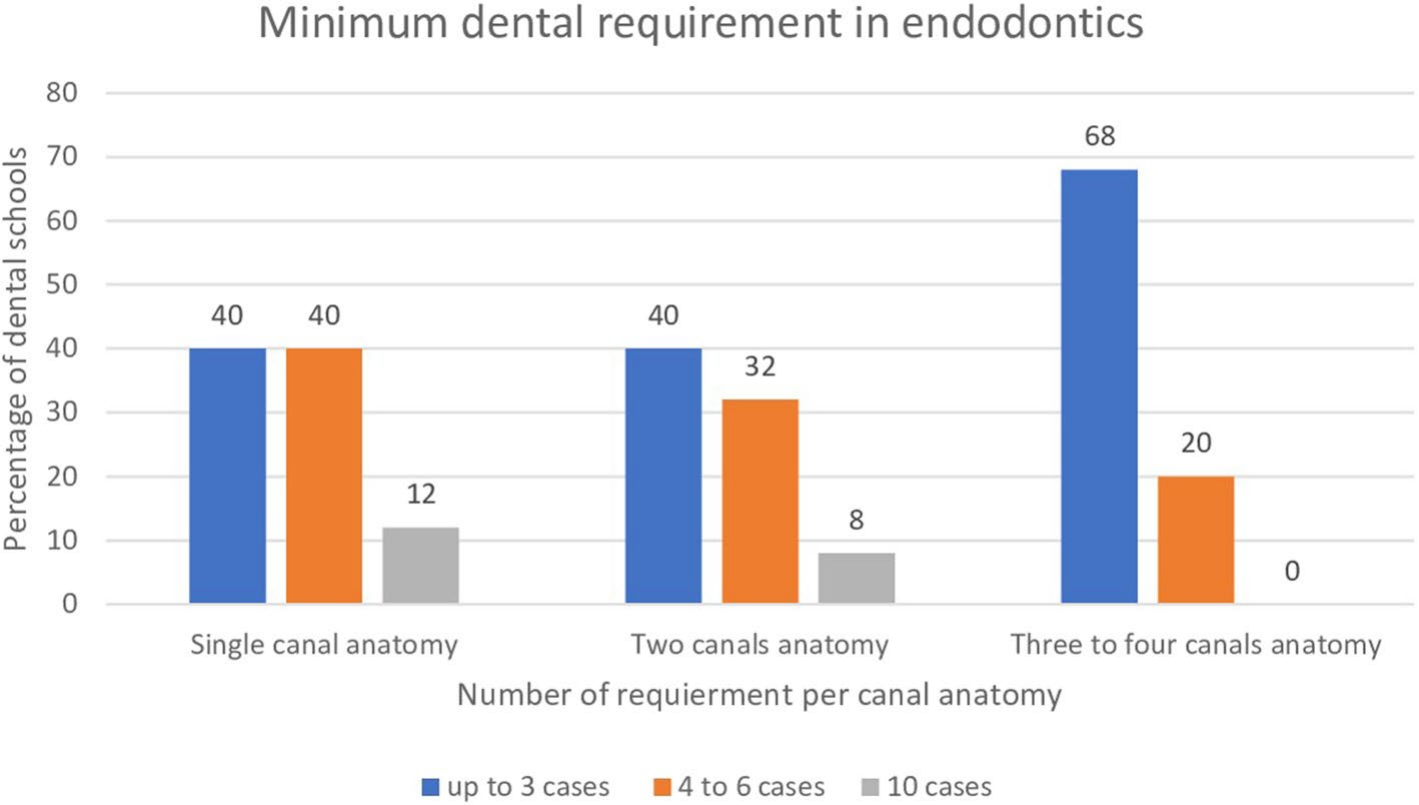
Percentage of dental schools according to the minimum number of root canal treatments required from dental students per canal anatomy

The majority of dental schools (92%) request students to complete a minimum number of endodontic requirements before graduation. Only two schools do not define the number nor the type of their requirements (8%). The minimum number of root canal treatments required before graduation is presented in Fig. 1. Single canal systems were frequently requested. The students usually have to complete up to three to six cases before graduation. Few dental schools ask their students to complete RCT of ten single canals (12%). Comparable findings were found in the two canals system in which few schools requested ten teeth (8%). Requirements involving three to four canal systems, however, were less than three teeth in most dental schools (68%) (Fig. 2). One school asked dental students to collect procedural points instead of defining the number or the type of procedures. In this model, the students will aim to collect procedural points that will allow them to reach the minimum number of points requested for their level of education in each dental specialty. The weight of the procedural points is adjusted according to the number of canals or the difficulty of the procedure. For example, multiple canals are given more weight than a single canal. Interestingly, one school additionally requested two cases of retreatment before graduation.

## Discussion

The objectives of the undergraduate endodontic program directors were to graduate competent practitioners who know the basic science of endodontics and who are competent in the diagnosis and management of simple endodontic cases. They also emphasized the importance of case selection to prevent malfeasance. Undergraduate students attend less than four credit hours of preclinical endodontic courses in which they are trained to do root canal treatment on natural teeth. In the clinical setting, most schools allowed students to do RCT on single, as well as, multiple canal systems except the second molar. Moreover, students are trained to do vital pulp therapy and RCT in simple and moderately difficult cases. The majority of dental students have a competency-based assessment in preclinical and clinical training. The students usually graduate after completing a minimum number of requirements in simple or moderately difficult cases. Unfortunately, the students are not consistently using the endodontic difficulty assessment forms and not signing a special endodontic treatment consent.

The endodontic undergraduate objectives are aligned with the European Society of Endodontology (ESE) and the Australian and New Zealand Academy of Endodontists undergraduate endodontic curriculum guidelines [37, 38]. The ESE and ADEE assume that dental students should be trained to be competent in RCT of uncomplicated single and multi-rooted teeth [5, 39, 40]. The Ministry of Health in Saudi Arabia expects qualified general dentists to be competent in RCT of anterior teeth, and mandates they refer treatment of posterior teeth to the endodontist after performing only necessary emergency intervention [41]. However, Saudi students are being trained to do RCT on all teeth types except for second molars. Moreover, the Saudi students are trained to treat cases of minimal to moderate level of complexity. Several studies found that undergraduate students were least confident in treating the molars among other teeth groups [31–33, 35, 42, 43]. Interestingly, the quality of RCT done in the Saudi public health sector was better than in the Saudi private sector and even better than some of the internationally reported findings [10]. One possible reason is that the general dentist in the Saudi public sector is more likely to follow the Ministry of Health recommendation and refer posterior teeth to an endodontist. The quality of RCT was reported to be significantly lower in molars [13]. The same finding was also reported studying the quality of RCT among undergraduate dental students [39, 40, 44]. Therefore, RCT of molars can become the objective of graduate training in the absence of sufficient time or resources to achieve satisfactory results in undergraduate training.

The Canadian and American endodontic case difficulty assessment form was developed to help dental practitioners to evaluate case complexity which will help them decide whether to treat or refer the case. Cases of high difficulty were defined as being clinically challenging for the most experienced practitioners. RCT of second molars and retreatments were regarded as high in difficulty [45]. The students were allowed to treat second molars in 36% of dental schools and perform retreatment in 68% of the dental schools. Also, the students were allowed to engage in the treatment of moderately complex cases. Case selection was one of the objectives of the undergraduate endodontic training. However, 40% of dental schools did not use any difficulty assessment form in clinical training. Tanalp et al. suggest that students are less confident in treating complex cases because they tend to refer these cases to postgraduate residents [35]. However, students perceived endodontic treatment as one of the most stressful dental procedures [46]. Because the endodontic procedure includes many steps such as local anesthesia administration, rubber dam isolation, and caries removal [46]. In addition to the endodontic access and root canal negotiation that requires multiple radiographs for assessment and completion of RCT visit. The management of patient anxiety and pain, post-operative pain, and endodontic flare-up may add another burden to undergraduate students [35]. Observation studies found that the quality of RCT and the endodontic treatment outcomes were less predictable in complex cases [35, 40, 44, 47, 48]. Therefore, it's probably best to refer these challenging cases to a specialist who can conveniently dedicate expertise, time, and resources to manage and follow these cases closely.

### The students

Competency-based education focuses on preparing dental students for professional life after graduation [49–52]. The laboratory practical exams, clinical competencies, and procedural requirements are methods used to assess competency [53]. The majority of dental schools in Saudi Arabia require students to pass a clinical competency exam and to complete a minimum number of procedural requirements. In a clinical competency exam, students carefully select their competency cases to pass the test. Then the faculty will assess the dental student's ability to provide adequate RCT independently [53]. The quality of RCT will be assessed according to international treatment standards. Therefore, students during this process will carefully practice case difficulty assessment and will realize the importance of case selection. Standardization of clinical circumstances can be improved by providing a clear description of suitable clinical cases [54]. For example, the clinical competency exam should be on an uncomplicated single canal in the anterior region.

The number of procedural requirements is different among Saudi dental schools. Currently, there is no sound evidence that supports the need to repeat a clinical dental procedure a certain number of times before approaching competency [55]. ESE used to recommend twenty natural teeth including extracted teeth as a minimum requirement for dental graduation [56]. Experiential learning theory can be used to support the importance of procedural requirements because it suggests that students learn best from experience [57]. Moreover, Self-efficacy was a positive predictor of dental academic performance [58]. The dental students' perception of self-efficacy was connected to their clinical experience [59]. Furthermore, students' endodontic self-efficacy improved as they performed more RCT [43]. However, those students endodontic self-efficacy declined in molars and retreatments [43].

Unexpectedly, Chamber observed that the learning curve in dental school was flat [55]. The possible explanation was that students aimed to perform best to pass grading criteria rather than gain dental experience. Moreover, he criticized the arbitrary practice of setting the procedural requirements numbers [55]. There is a need for further studies that explore the benefits of this practice and that guide educators in the decision-making process. Procedural requirements were regarded as an unethical practice [53]. Moreover, dental students found procedural requirements stressful [60]. Dental students in Saudi Arabia reported a high level of stress during their clinical training [61, 62]. Endodontic procedure particularly was one of the most stressful clinical events in dental training [46]. Thoughtfully, endodontic education guidelines in Australia and New Zealand extended the duration of comprehensive inter-disciplinary clinical care to at least two years [38]. However, the exact number and type of teeth were not determined since it depends on patient availability [38]. Hence, emphasizing the duration of clinical training rather than the number and type of teeth could be beneficial and less stressful for dental students [38].

Few dental schools did not select practical exams or clinical requirements as assessment criteria. Alternatives and adjunctive techniques that were suggested to assess competency are portfolios, objective structured clinical examination (OSCE), triple jump exams, and longitudinal and daily evaluation [54]. However, these assessment techniques were not investigated in this study.

Dental schools in Saudi Arabia don’t use endodontic consent for treatment consistently. General treatment consent might not clarify the possible emerging endodontic complications such as failure to achieve local anesthesia, post-operative pain, ledge, or broken instrument. Moreover, endodontic treatment consent is a valuable tool that would reinforce students' need for careful case assessment and treatment discussion with the patient. Finally, endodontic consent would set the treatment expectations for the patients and allow them to make informed dental decisions [63].

Most of the endodontic topics were covered which was similar to the findings of the United Kingdom and Spain [36, 64]. Fortunately, 96% of preclinical training was on natural teeth. Preclinical training on natural teeth is recommended since it's more challenging and realistic [65, 66]. Alternative options to natural teeth have been explored in the literature [65, 66]. The public and private schools had a similar approach to the instructional design of undergraduate endodontics in Saudi Arabia. One possible explanation is that the sample size for both of these schools was small to detect actual differences between them. Since the statistical findings were highly susceptible to type II error, the results have to be carefully interpreted.

The development of national guidelines for undergraduate endodontic education is recommended since it will improve the alliance among Saudi dental schools. Moreover, national guidelines would combine up-to-date evidence in best practices in education and endodontics. These guidelines will help undergraduate program directors to make key decisions that will enhance the training and performance of dental graduates. Finally, further research is needed to explore the best teaching and assessment methods to ensure endodontic competency, specifical topics such as the learning curve in endodontics and the requirements number.

## Conclusion

The undergraduate endodontic objectives are aligned with European endodontic guidelines. Saudi students are trained to do vital pulp therapy and RCT on mild to moderately complex cases. Procedural requirements and competency clinical exams are used to assess competency in the majority of dental schools. Endodontic treatment consent and difficulty assessment form are not consistently used. The development of national guidelines for undergraduate endodontic education is recommended.

## Supplementary Information

**Additional file 1.**

**Additional file 2. **

## Data Availability

All data generated or analysed during this study are included in this published article [and its supplementary information files].

## Abbreviations

RCT: Root canal treatment
CODA: The Commission of Dental Accreditation
ADEE: Association of Dental Education in Europe
ESE: European Society of Endodontology
OSCE: Objective structured clinical examination
